# Estradiol-mediated enhancement of the human ectocervical epithelial barrier correlates with desmoglein-1 expression in the follicular menstrual phase

**DOI:** 10.3389/fendo.2024.1454006

**Published:** 2024-10-08

**Authors:** Frideborg Bradley, Alexandra Stern, Mathias Franzén Boger, Zaynab Mousavian, Olga Dethlefsen, Vilde Kaldhusdal, Julie Lajoie, Kenneth Omollo, Sofia Bergström, Anna Månberg, Peter Nilsson, Joshua Kimani, Adam D. Burgener, Annelie Tjernlund, Christopher Sundling, Keith R. Fowke, Kristina Broliden

**Affiliations:** ^1^ Department of Medicine Solna, Division of Infectious Diseases, Karolinska Institutet, Department of Infectious Diseases, Karolinska University Hospital, Center for Molecular Medicine, Stockholm, Sweden; ^2^ National Bioinformatics Infrastructure Sweden, Science for Life Laboratory, Stockholm University, Stockholm, Sweden; ^3^ Department of Medical Microbiology and Infectious Diseases, University of Manitoba, Winnipeg, MB, Canada; ^4^ Department of Medical Microbiology, University of Nairobi, Nairobi, Kenya; ^5^ Partners for Health and Development in Africa, Nairobi, Kenya; ^6^ Division of Affinity Proteomics, Department of Protein Science, SciLifeLab, KTH Royal Institute of Technology, Stockholm, Sweden; ^7^ Center for Global Health and Diseases, Department of Pathology, Case Western Reserve University, Cleveland, OH, United States; ^8^ Department of Obstetrics and Gynecology, University of Manitoba, Winnipeg, MB, Canada; ^9^ Department of Community Health Sciences, University of Manitoba, Winnipeg, MB, Canada

**Keywords:** estradiol, progesterone, desmoglein-1, ectocervix, menstrual cycle, protein marker, gene expression, sexually transmitted infections

## Abstract

**Background:**

The cervicovaginal epithelial barrier is crucial for defending the female reproductive tract against sexually transmitted infections. Hormones, specifically estradiol and progesterone, along with their respective receptor expressions, play an important role in modulating this barrier. However, the influence of estradiol and progesterone on gene and protein expression in the ectocervical mucosa of naturally cycling women is not well understood.

**Methods:**

Mucosal and blood samples were collected from Kenyan female sex workers at high risk of sexually transmitted infections. All samples were obtained at two time points, separated by two weeks, aiming for the follicular and luteal phases of the menstrual cycle. Ectocervical tissue biopsies were analyzed by RNA-sequencing and *in situ* immunofluorescence staining, cervicovaginal lavage samples (CVL) were evaluated using protein profiling, and plasma samples were analyzed for hormone levels.

**Results:**

Unsupervised clustering of RNA-sequencing data was performed using Weighted gene co-expression network analysis (WGCNA). In the follicular phase, estradiol levels positively correlated with a gene module representing epithelial structure and function, and negatively correlated with a gene module representing cell cycle regulation. These correlations were confirmed using regression analysis including adjustment for bacterial vaginosis status. Using WGCNA, no gene module correlated with progesterone levels in the follicular phase. In the luteal phase, no gene module correlated with either estradiol or progesterone levels. Protein profiling on CVL revealed that higher levels of estradiol during the follicular phase correlated with increased expression of epithelial barrier integrity markers, including DSG1. This contrasted to the limited correlations of protein expression with estradiol levels in the luteal phase. *In situ* imaging analysis confirmed that higher estradiol levels during the follicular phase correlated with increased DSG1 expression.

**Conclusion:**

We demonstrate that estradiol levels positively correlate with specific markers of ectocervical epithelial structure and function, particularly DSG1, during the follicular phase of the menstrual cycle. Neither progesterone levels during the follicular phase nor estradiol and progesterone levels during the luteal phase correlated with any specific sets of gene markers. These findings align with the expression of estradiol and progesterone receptors in the ectocervical epithelium during these menstrual phases.

## 1 Introduction

Female sex hormones influence susceptibility to sexually transmitted infections (STIs) by affecting epithelial barrier integrity and immune responses in the genital tract mucosa (1, 2). The main sex hormones in premenopausal women are estradiol and progesterone, which govern cyclic changes in the genital tract during the menstrual cycle. Their physiological role is not only regulated by their concentration, but also by the corresponding receptor expression which can vary during the menstrual cycle and across the genital tract mucosa. The ectocervical epithelium normally expresses the estrogen receptor (ER), but not the progesterone receptor (PR), in the follicular phase. During the luteal phase, ER and PR are only weakly expressed in the ectocervical epithelium (3, 4). In contrast, both ER and PR are highly expressed in the transformation zone of the human uterine cervix (5). Among many effects, these hormones (and their synthetic analogs in hormonal contraceptives) influence the expression of epithelial junction proteins (EJPs) within the cervicovaginal epithelium (6–9). Similar effects have been observed in nonhuman primates, (10, 11) mice, (12–15) humanized mice, (16, 17) genital epithelial cell lines, (18, 19), and an experimental human tissue model (20).

Postmenopausal women, with naturally low levels of both estradiol and progesterone, exhibit thinner vaginal (21) and ectocervical (22) epithelium. Treatment with estradiol-containing products in these women improves the vaginal mucosa and relieves vaginal discomfort. At the molecular level, this treatment can increase the expression of the adherens junction protein E-cadherin (21), the desmosome protein desmoglein-1 (DSG1) and other proteins involved in epithelial development or repair of human vaginal tissue (23). In a nonhuman primate model, treatment with various estrogen compounds has proven effective against transmission of vaginal simian immunodeficiency virus (24, 25) and restores genital epithelial barrier integrity (11). In an ectocervical tissue model, experimental infection with the human immunodeficiency virus (HIV) was more efficient in tissue samples from postmenopausal women than in samples from premenopausal women (21). Treatment with vaginal estradiol cream mitigated this increased HIV susceptibility in the postmenopausal samples.

Other proposed mechanisms for how female sex hormones influence the genital mucosa include a shift in cervicovaginal microbiome composition, as exemplified in adolescent girls (26), and effects on local immune cell repertoire and function [reviewed in (2)]. We previously reported that expression of E-cadherin (6) and DSG1 (8) in human ectocervical tissue from premenopausal women correlated positively with systemic estradiol levels. However, a more comprehensive approach to analyzing human ectocervical mucosal gene and protein expression in relation to cycling estradiol and progesterone levels has, to the best of our knowledge, not yet been performed. Information generated in this manner will improve our understanding of the structural integrity and immunological features of the genital mucosa in relation to STI susceptibility.

In this study, we investigate the impact of endogenous estradiol and progesterone levels on the ectocervical mucosa of premenopausal Kenyan female sex workers. These women were specifically chosen due to their regular menstrual cycles and their representation of a population at high risk of STIs. Our findings demonstrate how the expression of specific epithelial barrier markers varies in response to sex hormone levels. Notably, these results align with ectocervical hormone receptor expression at this specific site in the female reproductive tract, providing a mechanistic insight into our observations.

## 2 Materials and methods

### 2.1 Study participants and sample collection

The samples were derived from a longitudinal study performed in the Pumwani Sex Worker cohort in Nairobi, Kenya (8, 27). Briefly, the inclusion criteria for the overall study were being active in self-reported sex work, aged 18–50 years, no prior hysterectomy, not pregnant or breastfeeding, and negative for HIV, *Chlamydia trachomatis*, *Neisseria gonorrhoeae*, syphilis, and *Trichomonas vaginalis* infection at enrollment. For the present study, we included only samples from women who had been pre-screened for having regular menstrual cycles and did not use hormonal contraceptives. Written, informed consent was obtained from all study participants. Ethical approval was granted by the Regional Ethical Review Board in Stockholm, Sweden, the University of Manitoba, Canada, and the Kenyatta National Hospital/University of Nairobi, Kenya.

At enrollment and during the two sampling study visits, testing was performed for the following: 1) bacterial vaginosis (BV), based on the Nugent score of gram-stained smears; 2) *Chlamydia trachomatis* and *N. gonorrhoeae*, based on polymerase chain reaction screening of urine samples using a Roche AMPLICOR kit (Pleasanton, NJ, USA); 3) syphilis, using a rapid plasma reagin serologic test (Macro-Vue Rapid Plasma Reagin test, Becton Dickinson, NJ, USA); and 4) *T. vaginalis*, based on saline microscopy of vaginal swab specimens. HIV serological testing was performed at enrollment, 6 weeks following the first visit, and 3–6 months after study completion using a rapid test (Determine, Inverness Medical, Japan). Participants also completed a demographic and behavioral questionnaire.

Strict measures, such as text-message reminders and repeated on-site detection of prostate-specific antigen, were applied to promote adherence to sexual abstinence for 2 weeks following each sample collection (27). After the first sampling aiming for the luteal phase (LUT), study participants were monetarily compensated for a 4-week period of abstinence to enable healing, as previously described (27). Two weeks into this period, the second sampling aiming for the follicular phase (FOL), was performed.

At each of the two study visits, venous blood and genital samples were collected. Briefly, 2 mL of sterile phosphate-buffered saline was flushed into the vaginal cavity and aspirated from the posterior fornix region. The lavage fluid was centrifuged to remove cellular debris, and the supernatant (referred to as cervicovaginal lavage, CVL) was aliquoted. Two 3-mm^2^ ectocervical biopsies were collected from the superior part of the ectocervix by a trained gynecologist using Schubert biopsy forceps (model ER058R, Aseculap, Germany). Biopsy tissue was snap-frozen in liquid nitrogen (for subsequent imaging analysis) or placed immediately in RNAlater (for subsequent RNA-sequencing analysis). All mucosal and plasma samples were stored at –80°C.

### 2.2 Timing of sampling and plasma hormone analysis

The first sample included in this study was obtained during the LUT phase of the menstrual cycle. Plasma levels of estradiol and progesterone during the LUT phase were measured using the Milliplex Map Steroid/Thyroid Hormone Magnetic Bead Panel (Millipore, Merck, Darmstadt, Germany). The lower limits of detection (LLDs) for estradiol and progesterone were 20 pg/mL and 0.09 ng/mL, respectively. Values below this were reported as “below LLD,” but for statistical analysis were assigned a value of approximately ½ of LLD: 10 pg/mL for estradiol and 0.05 ng/mL for progesterone.

The second sample was obtained during the FOL phase. Plasma estradiol and progesterone levels in FOL samples were measured using electrochemiluminescence immunoassays (Roche Diagnostics) at the accredited Karolinska University Laboratory, Stockholm (8). The LLDs for estradiol and progesterone were 22 pg/mL and 0.05 ng/mL, respectively. Values <22 pg/mL (estradiol) and <0.05 ng/mL (progesterone) were reported as “below LLD,” but for statistical analysis were assigned values of 22 pg/mL and 0.05 ng/mL, respectively. The different analytical methods and LLD principles applied for the two phases were taken into account by avoiding direct comparisons of experimental data between the phases.

Correlations between estradiol and progesterone at each visit were determined using Spearman’s correlation test. SPSS (version 28; IBM) was used for these analyses, and p-values <0.05 were considered significant. For the reader’s convenience, we will henceforth address the FOL phase first, followed by the LUT phase.

### 2.3 RNA extraction, preparation of libraries for RNA-sequencing analysis, and data analysis

Tissue samples in RNAlater from both the FOL and the LUT phases were prepared for RNA-sequencing (8). A partially overlapping cohort of samples from the FOL phase, but not the LUT phase, has been previously published (6, 8, 28). Briefly, the biopsies were thawed, placed in RLT Plus Lysis buffer (QIAGEN, Hilden, Germany), and homogenized using a TissueLyzer II machine (QIAGEN). An AllPrep DNA/RNA Mini Kit (QIAGEN) was used for RNA isolation and purification, and RNA quality was assessed by determining the RNA integrity number.

Library construction was performed as previously described (8). Briefly, a TruSeq mRNA SEq Library Prep kit (Illumina, San Diego, USA) was used to convert mRNA to cDNA libraries. Libraries (barcoded) were pooled and sequenced using NS550 SR 75-bp (for FOL samples) or Novaseq 6000 PE 150-bp (Illumina) (for LUT samples). Tissue samples from both FOL and LUT phases exhibited RNA integrity number values >7. However, for the current study, we performed a new alignment and generated new counts for FOL data using the Ensembl genome and annotations to generate more accurate comparisons with the new LUT phase set. For the LUT samples, Bcl files were demultiplexed and converted to fastq using the bcl2fastq v2.20.0.422 program and subsequently trimmed using fastp 0.21.0 to remove Illumina adapter sequences. STAR 2.7.9a (29) was used to index the human reference genome (hg38/GRCh38) and align the resulting fastq files. Mapped reads were then counted for annotated exons using featureCounts v1.5.1 (30). The gene annotations (Homo_sapiens.GRCh38.101.gtf) and reference genome were obtained from Ensembl. Gene expression data were analyzed using EdgeR v4.0.16 (31). Genes with low expression were filtered out using filterByExpr function, while genes with a count per million of ≥10 in ≥70% of samples were retained. Trimmed mean of M-values normalization was used to correct for the library size differences and compositional variations (32), and normalized count data were further log_2_ transformed. RNA-sequencing data for FOL samples were deposited previously in the Gene Expression Omnibus (GEO) data repository, and all new data has been added to the same superseries, as further described in the Data Statement Availability section.

### 2.4 Weighted gene co-expression network analysis

We performed weighted gene co-expression network analysis (WGCNA) using the WGCNA R package (33) to construct a weighted gene co-expression network using the 5000 most variable genes in our RNA-sequencing dataset from both the FOL and the LUT phase visits. We used the *adjacency* function in this package, with the input parameters of “bicor” correlation and “signed hybrid” network type. Biweight midcorrelation (bicor) is a median-based correlation that enhances the robustness of the analysis (34). In the signed hybrid network, only positive correlation links were retained, while negative correlations were disregarded. Using the *pickSoftThreshold* function in the WGCNA package, we identified the optimal power parameter value to create a scale-free network, a commonly observed characteristic in biological networks. Following network construction, hierarchical clustering was performed using the *cutreeDynamic* function of the WGCNA package to cluster the network into modules. The clustering parameters were deepSplit=2 and minClusterSize=40, with other parameters set to their default values. To reduce the number of identified modules, closely related modules were merged. Gene expression profiles within each module were summarized using module eigengenes. In the *moduleEigengenes* function of the WGCNA package, the module eigengenes for each module was computed as the first principal component of the expression data. To identify modules associated with estradiol level, progesterone level, or both, we computed the correlation between each module eigengenes and both estradiol and progesterone levels.

### 2.5 Preservation analysis of modules for the follicular and luteal phases

We used gene expression data from the LUT phase to validate whether modules derived from FOL phase data were preserved. Among the 5000 most variable genes in the LUT dataset, we specifically selected genes that overlapped with the FOL dataset (n=4031). After constructing a co-expression network with the FOL data set, using the overlapping genes, we identified gene modules representing the transcriptional landscape of the FOL state. To quantitatively assess the degree of module preservation between the FOL and the LUT phases, we employed Z summary statistics calculated for each module separately using the *modulePreservation* function in the WGCNA R package. Preservation of a module was considered moderate if the Z-statistic was 2–10 and strong if it was >10 (35).

### 2.6 Regression analysis to identify differentially expressed genes associated with estradiol and progesterone levels

To identify differentially expressed genes (DEGs) affected by female sex hormone levels at both the FOL and LUT phase visit, we used the edgeR package to determine the associations of genes with estradiol and progesterone levels, adjusting for BV status (BV, intermediate, or normal). BV status was added as a variable in the model because the composition of the cervicovaginal microbiome has been shown to significantly affect the ectocervical transcriptome (36). Quasi-likelihood negative binomial generalized log-linear models were fitted to our data, and gene-wise statistical tests for a given coefficient (estradiol or progesterone) were run to identify significant genes. P-values were adjusted for multiple testing using the Benjamin and Hochberg procedure, and genes with false discovery rate–adjusted (FDR-adj) p-values <0.05 were considered DEGs.

### 2.7 Functional enrichment analysis

To gain insights into the biological functions and pathways associated with the modules of interest, we performed functional enrichment analysis using the enrichR R package. We performed functional enrichment analysis of significant modules and included only genes with a module membership >0.5 and a p-value module membership <0.05. We used the Gene Ontology (GO) (37), Kyoto Encyclopedia of Genes and Genomes (KEGG) (38), and Transcriptional Regulatory Relationships Unraveled by Science-based Text (TRRUST) (39) databases for functional annotation and enrichment analysis.

### 2.8 Antibody-based protein profiling of genital secretions

An antibody-based protein profiling assay encompassing 74 target proteins, represented by 90 antibodies (1-3 antibodies per protein), was performed on CVL for both the FOL and the LUT phase visit (**Supplementary Table 1**). The target proteins were selected based on their association with inflammation, HIV resistance and sex hormones in the female genital tract (7, 40–45). This panel has been used previously with a partially overlapping cohort of samples (8, 28, 36). A previously published protocol for protein profiling was followed (40). Briefly, preselected antibodies from the Human Protein Atlas (46) were attached to color-coded beads and mixed with biotinylated CVL samples. A streptavidin-conjugated fluorophore was added to enable detection using a Flexmap 3D instrument (Luminex Corp., Austin, TX, USA). Fluorescence intensity in arbitrary units was used to evaluate binding events. The protein profiling data was log10 transformed and normalized to diminish the effects of time delay during instrument readout and potential differences between plates.

### 2.9 *In situ* immunofluorescence staining of DSG1, claudin-1, ZO-1, and E-cadherin in ectocervical epithelium

To analyze protein expression and spatial distribution of selected EJPs from the FOL phase visit, *in situ* staining was conducted on cryopreserved ectocervical biopsy sections with a thickness of 8 µm. We previously performed *in situ* staining of DSG1, claudin-1, and E-cadherin in a cohort largely overlapping with the current cohort (6, 8, 28). Since then, we have developed a new bioimage analysis workflow with additional measurements, as described in the next section.

Staining for E-cadherin was performed as previously described (6). Briefly, tissue sections were incubated with mouse anti-human E-cadherin primary antibody, followed by the addition of Alexa Fluor 488-conjugated donkey anti-mouse IgG secondary antibody (**Supplementary Table 2**). To perform triple staining of DSG1, claudin-1, and zonula occludens 1 (ZO-1), sections were first mounted on SuperFrost^®^ Gold Plus slides (Menzel Gläser, Thermo Fischer Scientific, VWR International AB, Kista, Sweden). The slides were air-dried for 1 hour, fixed in concentrated acetone (VWR International AB, Stockholm, Sweden) for 15 minutes, and then air-dried for 10 minutes. Staining was performed by incubation with primary monoclonal antibodies, followed by incubation with secondary fluorophore-conjugated antibodies (**Supplementary Table 2**). Negative controls were incubated without primary antibody, and all slides were counterstained with 4’6-diamidino-2-phenylindole (Invitrogen, Thermo Fischer Scientific, Stockholm, Sweden). After each step, the slides were washed in PBS (1x), HEPES (1%) (HyClone, Nordic Biolabs, Täby, Sweden), and saponin (0.1%) (Sigma-Aldrich, Solna, Sweden). After the final washing step, the slides were mounted using Dako fluorescence mounting buffer (Agilent, Santa Clara, CA, USA) and scanned as digital images using a Pannoramic 250 Flash Slide Scanner at 20× magnification (3DHISTECH Ltd, Budapest, Hungary).

### 2.10 Bioimage analysis of DSG1, claudin-1, ZO-1, and E-cadherin in ectocervical epithelium

To assess *in situ* staining of EJPs, Caseviewer (version 2.4, 3DHISTECH Ltd) was used to manually annotate 2–6 regions of interest (ROIs) per sample. To ensure objectivity, annotations of all ROIs were performed under blinded conditions. The average ROI data for each sample was used for statistical analysis. The epithelial compartment was marked in FIJI (v.153c) (47), and the net structures formed by DSG1, claudin-1, ZO-1, and E-cadherin protein strands were enhanced in MATLAB (vR2020b, MathWorks, Natick, MA, US) using a contrast-independent approach that enhanced curve-linear structures (48) (**Supplementary Figure 1**). The epithelium was segmented into three layers based on spatial expression of the EJP strands: the superficial layer, located apically toward the vaginal lumen and lacking EJP staining; the intermediate layer, which expressed EJP; and the basal layer, located toward the basal membrane and lacking EJP staining (**Supplementary Figure 1**). Mean fluorescence intensity (MFI) was calculated within the entire epithelial compartment and the intermediate layer for each EJP and was used as a proxy of protein expression. The height of the epithelial compartment was calculated using a Euclidean distance-transform image where the distance (intensity) from each point on the superficial border to the basal border and back was calculated. The net-like structure of each EJP was defined as either intact or fragmented, based on the connectivity of the expressed protein strands (**Supplementary Figure 1**). Any break greater than one pixel (0.325 µm) was regarded as sufficient to allow for viral passage, thereby classifying the net as fragmented. A model was developed to assess the theoretical accessibility of external virus exposure. This involved using a digital flooding technique, starting at the apical ectocervical border and moving toward the basal membrane, in which an intact net served as the principal protective barrier. Consequently, an accessible region was generated for each EJP (**Supplementary Figure 1**). Finally, to analyze epithelial stability, the intermediate layer was separated into intact and fragmented regions by performing a watershed transformation of all holes in the intact net (**Supplementary Figure 1**). The percentages of epithelial coverage of the intact and accessible regions were computed for each EJP.

### 2.11 Correlation analysis of sex hormones with protein expression and bioimaging results

Spearman’s correlation analysis was used to assess correlations between plasma levels of estradiol and progesterone and protein expression, as determined by antibody-based protein profiling, as well as all parameters acquired by image analysis for each protein (DSG1, E-cadherin, claudin-1, and ZO-1). All samples with values for these variables were included in the analyses. Given our targeted approach, based on pre-selected proteins and parameters, no multiple comparison corrections were performed, and unadjusted p-values <0.05 were considered significant. SPSS (version 28; IBM) was used for correlation analysis of all image analysis parameters, and Prism (version 9.4.0) was used for correlation analysis of the protein profiling data.

## 3 Results

### 3.1 Sociodemographic data and clinical characteristics of study participants

**Figure 1:**
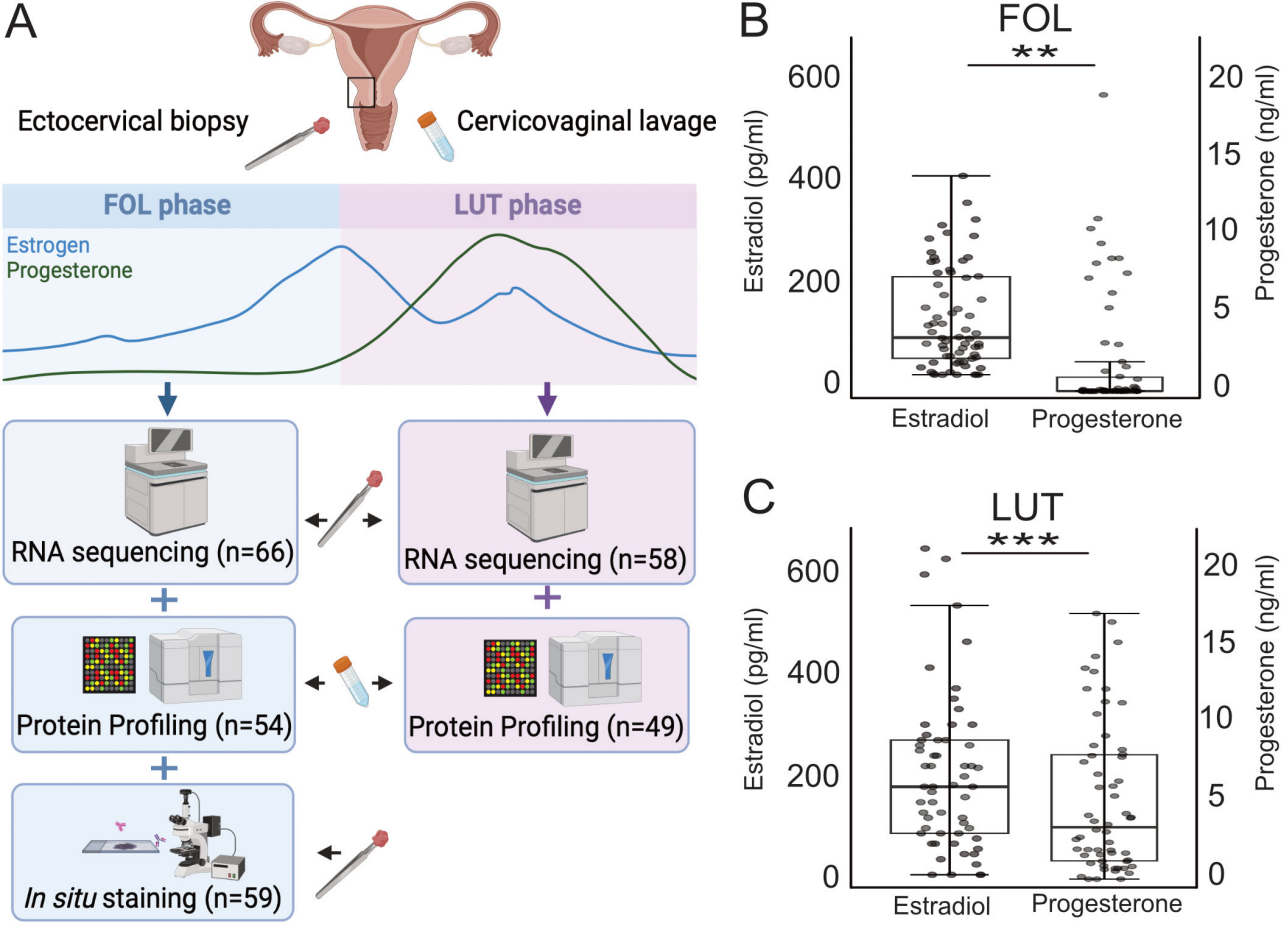
Overview of the study setup and systemic hormone levels at the FOL and LUT visits. **(A)** In this study, RNA sequencing was performed on ectocervical biopsies collected during the FOL (n=66) and LUT (n=58) phase of the menstrual cycle. Protein profiling was performed on CVL collected during the FOL (n=54) and LUT (n=49) phase and *in situ* immunofluorescence was performed on ectocervical biopsies collected during the FOL phase (n=59). Levels of estradiol and progesterone in plasma at the, **(B)** FOL visit (n=66), and at the, **(C)** LUT visit (n=58). The boxes show the IQR and the horizontal line within each box represents the median value. IQR, interquartile range. P-values <0.05 are considered significant (Mann-Whitney U test). *p <0.05, **p <0.01, ***p <0.001. FOL, follicular; LUT, luteal; CVL, cervicovaginal lavage. **(A)** is created with BioRender.com.

Samples of ectocervical tissue and CVL were obtained from Kenyan female sex workers (**Figure 1A**). Samples were obtained during both the FOL phase (n=66) and the LUT phase (n=58) of the menstrual cycle (**Table 1**). Samples collected at both times from the same woman were available for 54 study participants. The median age was 34 and 35 years, and median duration of sex work was 36 and 54 months in the FOL and LUT groups, respectively. The discrepancy was driven by the non-overlapping individuals and the phases will not be compared as such (**Supplementary Table 3**). The percentage of study participants positive for BV was 35% in the FOL group and 21% in the LUT group. One individual tested positive for *C. trachomatis* at both the FOL and LUT visits (although testing was negative at enrollment). All women remained HIV-seronegative for 3–6 months after completing the study.

**Table 1:** Characteristics of study subjects in the RNA sequencing analysis.

	FOL (n=66)	LUT (n=58)
	Number or median (range or %)	Number or median (range or %)
**Age,** years, median (range)	34 (20, 50)	35 (21, 50)
Months in sex work*, median (range)	36 (2, 372)	54 (4, 372)
Having a regular partner, No. (%)		
- Yes	40 (61%)	33 (57%)
**Years in school,** median (range)	10 (7, 21)	10 (5, 21)
Bacterial Vaginosis (BV; based on Nugent Score), No. (%)		
- BV	23 (35%)	12 (21%)
- Intermediate	17 (26%)	15 (26%)
- Normal	26 (40%)	30 (52%)
- Not available	0 (0%)	1 (2%)
Presence of STI**, No. (%)	1 CT (2%)	1 CT (2%)
**Self-reported days since onset of last menses,** median (range)	9 (3, 44)	21 (6, 31)
- Not available	4 (6%)	0 (0%)
Plasma hormone levels, median (range)		
*Estradiol, (pg/ml)*	94 (22, 405)	180 (10,640)
- Below LLD**	6 (9%)	4 (7%)
*Progesterone, (ng/ml)*	0.05 (0.05, 19)	3.4 (0.05, 17)
- Below LLD***	34 (52%)	3 (5%)

*The difference in sex work duration between the groups is due to 4 unique LUT samples with a higher median time compared to 12 unique FOL samples.

**Having an ongoing STI at time of enrolment (approximately 4 weeks prior to first sample visit) was an exclusion criterium for participating in the study. At each visit, testing was repeated for CT, NG, syphilis and Trichomonas vaginalis.

***LLD for estradiol was 20 pg/ml for the LUT visit and 22 pg/ml for the FOL visit.

****LLD for progesterone was 0.09 ng/ml for the LUT visit and 0.05 ng/ml for the FOL visit.

FOL, follicular; LUT, luteal; STI, sexually transmitted infections; CT, Chlamydia trachomatis; NG, Neisseria gonorrhoeae; LLD, lower limit of detection.

Samples from both the FOL and LUT phases were included to represent different estradiol and progesterone concentrations as previously established (49), and these levels are presented in detail (**Table 1**; **Figures 1B, C**). There was a significant positive correlation between estradiol and progesterone during both the FOL (r=0.44, p<0.001), and LUT (r=0.46, p<0.001) phases (**Supplementary Figure 2**).

### 3.2 Weighted gene co-expression network analysis revealed clusters of epithelial barrier and cell cycle regulation genes correlating with systemic estradiol levels

**Figure 2:**
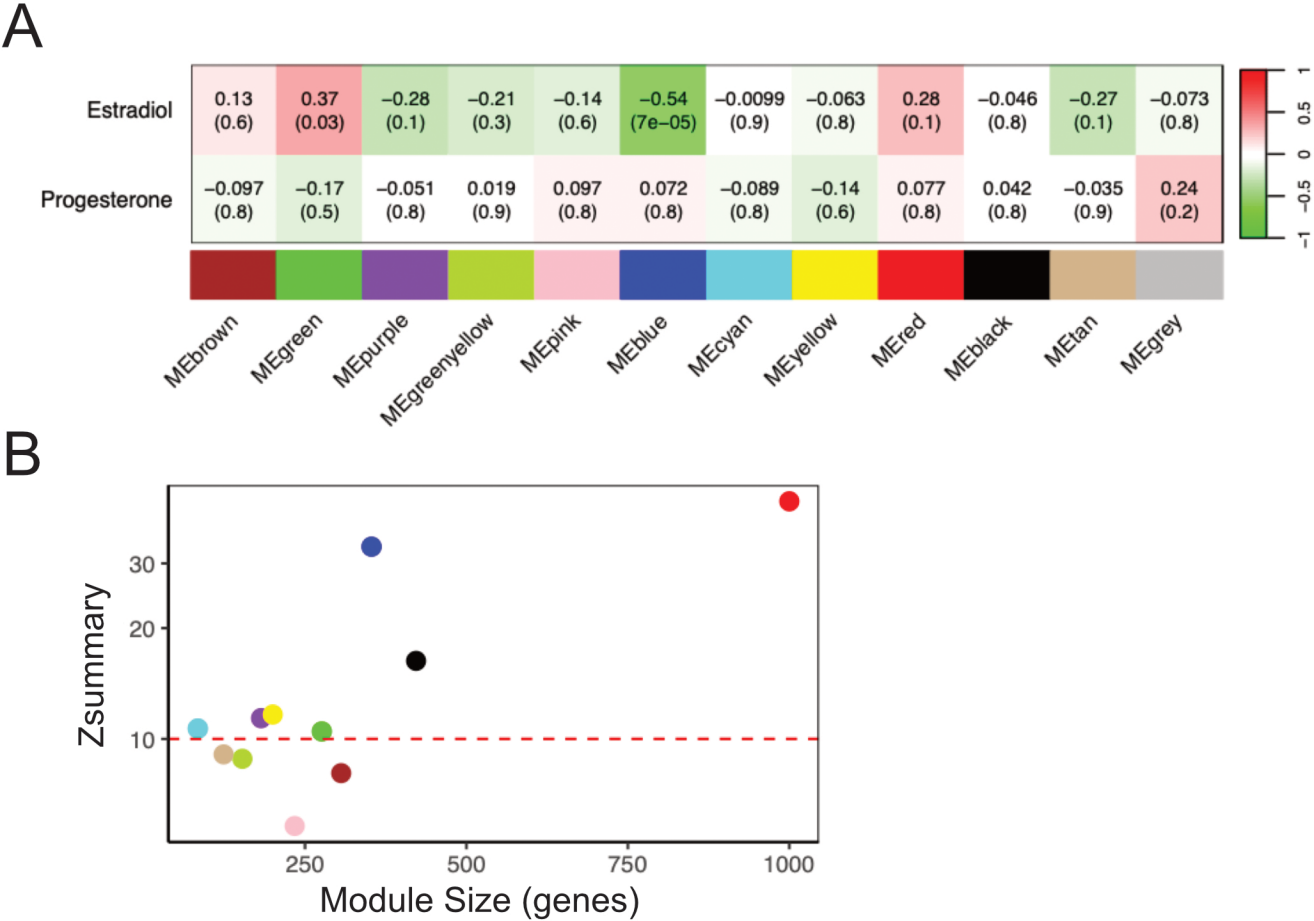
Gene Co-expression Network Analysis reveals clusters of genes correlated with systemic levels of estradiol. **(A)** The correlation between each module eigengene and the plasma levels of estradiol and progesterone in the FOL phase. The first value in each cell represents the Pearson correlation coefficient, and the value in parenthesis signifies the associated p-value. The strength of the association using Pearson correlation coefficient is also indicated by the color of the cell, green color representing a negative association and red color a positive association with serum levels. **(B)** Preservation analysis of co-expressed modules identified in the FOL phase in comparison to the LUT phase. Modules exhibiting a Zsummary score greater than 10, showed by the red dashed line in the plot, indicate strong preservation between the visits. FOL, follicular; LUT, luteal.

To provide a comprehensive understanding of the effects of female sex hormones on the transcriptome in the ectocervical epithelium, we performed RNA-sequencing of ectocervical tissue biopsies from both the FOL and LUT phase. A total of 14689 genes in the FOL phase and 13249 genes in the LUT phase were identified after filtering and normalization (**Supplementary Table 4**). Given biological functions are highly complex and depend on co-expression of genes, unsupervised clustering of RNA-sequencing data was performed using WGCNA. Gene clusters (modules) identified by WGCNA represent genes related to specific biological processes or phenotypic traits. We constructed such a network to analyze correlations of gene expression profiles across all samples from the FOL and LUT phase, respectively. After clustering and merging of close modules, we identified 11 distinct modules in the FOL phase samples, each assigned a different color (**Figure 2A**; **Supplementary Table 5**). The modules varied in size from 91 to 1998 genes and collectively consisted of 4779 genes, from a total of 5000 included genes. The remaining 221 genes that did not belong to a specific module were assigned to the grey module and excluded from further analysis. Next, we calculated the Pearson’s correlation between the module eigengenes (representing the expression diversity of all genes in the module) and estradiol or progesterone levels. The green module, consisting of 303 genes, demonstrated a significant positive correlation with estradiol levels (r=0.37, FDR-adj p=0.03), while the blue module, comprising 415 genes, displayed a significant negative correlation with estradiol levels (r=0.54, FDR-adj p=7E−05) (**Supplementary Table 5**; **Figure 2A**). To retain only genes strongly associated with each module, we filtered the modules by selecting genes with a module membership >0.5 and a p-value module membership <0.05. Genes in the filtered green module (206 genes) included several genes important for epithelial barrier function, such as *DSG1, CLDN17, FLG, RPTN, LORICRIN*, and several keratin family genes (*KRTDAP* and *KRT 2, 3, 6A–C, 7, 9, 10, 13, 14, 16, 33b*, and *76*) (**Supplementary Table 5**). The filtered blue module (203 genes) contained several genes associated with various types of cancers and cell cycle regulation, including *TTK, PLK1, LRG1*, and *CDK1* (**Supplementary Table 5**). No module was significantly associated with progesterone levels during the follicular phase (**Figure 2A**). Overall, these results indicate that estradiol levels, during the follicular phase, positively correlated with genes involved in epithelial barrier structure and function and negatively correlated with genes involved in cell cycle regulation.

After clustering 5000 of the most varying genes from the LUT phase samples into modules, we identified 9 distinct modules, each assigned a different color (**Supplementary Figure 3**; **Supplementary Table 5**). The modules varied in size from 70 to 2015 genes and collectively consisted of 4112 genes. The remaining 888 genes that did not belong to a specific module were assigned to the grey module and excluded from further analysis. No module was significantly correlated with neither estradiol nor progesterone in the LUT phase (**Supplementary Figure 3**).

### 3.3 Modules identified at the follicular phase visit were preserved at the luteal phase visit

To determine whether the modules identified in the FOL samples were preserved in the LUT samples, we performed a preservation analysis. As shown in **Figure 2B**, 4 modules exhibited Z-statistic values between 5 and 10, signifying moderate preservation, and 7 modules (including the green and blue modules) displayed Z-statistic values >10, reflecting strong preservation. These results indicate that gene co-expression patterns, regardless of if a module was significant or not, were similar for the majority of genes at both time points.

### 3.4 Epithelial barrier genes associated with higher estradiol levels using WGCNA were confirmed by regression analysis

**Figure 3:**
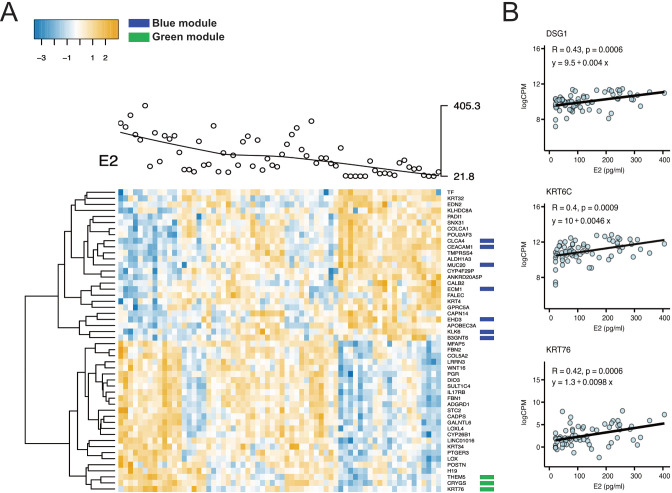
Correlations between gene expression patterns and plasma estradiol levels in the FOL phase. **(A)** Heatmap of the top 25 negatively and the top 25 positively associated DEGs (FDR-adj. p<0.05) with levels of estradiol in the FOL phase. Each gene is shown on the horizontal row and each sample on the vertical row. The blue color represents a gene expression below average and yellow color a gene expression above average. The green and dark blue colored boxes represent genes in the WCGNA-generated filtered green and blue modules, respectively. **(B)** Plasma levels of estradiol are depicted on the x-axis and log CPM on the y-axis for the genes *DSG1*, *KRT6C* and *KRT76* in the FOL phase. These genes were identified as associated with levels of estradiol both within the green module (using WCGNA) as well as using the regression analysis. Each dot represents a sample, and the thick black line the estimated regression line (y). R value, Pearson correlation coefficient; FDR adjusted p-value, from linear regression for estradiol term testing the null hypothesis that the estradiol coefficient is equal to zero (no effect). FOL, follicular; DEG, differentially expressed genes; FDR, false discovery rate; CPM, Counts per million; WCGNA, Weighted gene co-expression network analysis.

To further explore the association between estradiol and progesterone levels and ectocervical gene expression and to confirm the results generated by WGCNA, we constructed a regression model including estradiol level, progesterone level, and BV status (a possible confounding factor) as independent variables. The possible confounder “age” did not influence the regression model for the FOL or LUT phase and was therefore not included in the model (**Supplementary Table 6**). We found that 314 genes were positively associated with estradiol levels, and 447 genes were negatively associated with estradiol levels in the FOL phase (FDR-adjusted p<0.05 for both) (**Supplementary Figure 4**; **Supplementary Table 6**). The top DEGs positively correlated with estradiol in the FOL phase included genes critical for epithelial barrier function (notably, members of the keratin family [e.g, *KRT76*, *KRT34*]) and stabilization of collagen (e.g, *COL5A2, LOX, LOXL4*) (**Figure 3A**). By contrast, the top DEGs negatively correlated with estradiol included the cysteine protease *CAPN14* (a protein involved in epithelial barrier disruption), the serine protease *TMPRSS4*, and two oncogenes (*ALDH1A3*, *KLK6*) (**Figure 3A**) (50–52).

Three genes were positively associated with progesterone, and one gene, a metalloproteinase (*MMP11*), was negatively associated with progesterone in the FOL phase samples (**Supplementary Table 6**). There was no overlap between genes associated with estradiol versus progesterone. No significant correlation was found between estradiol and gene expression in the LUT phase, neither was any correlation found between progesterone and gene expression in this phase (**Supplementary Table 6**).

Genes from the two WGCNA-generated modules (significantly associated with estradiol) were compared with the DEGs associated with estradiol from the regression analysis in the FOL phase samples. In the filtered green module, 106 (52%) and 40 (20%) of the 206 genes were positively associated with estradiol in the unadjusted (p<0.05) and the adjusted (FDR-adjusted p<0.05) regression analyses, respectively (**Supplementary Tables 5, 6**). In total, 187 genes (90%) in the filtered green module showed a positive log2 fold change (FC) in the regression analysis (**Supplementary Tables 5, 6**). Several epithelial barrier genes in the green module, including *DSG1, KRT76*, and *KRT6C*, were confirmed to be positively correlated with estradiol levels in the regression analysis (**Figure 3B**). A similar pattern was observed in the filtered blue module. Of the 203 genes in this module, 175 (86%) and 86 (42%) were negatively associated with estradiol in the unadjusted (p<0.05) and the adjusted (FDR-adj p<0.05) regression analyses, respectively (**Supplementary Tables 5, 6**). All genes in the blue module exhibited a negative log2FC. Overall, these results indicate that despite different methods of analysis and the inclusion of BV status in our regression analysis (but not in the WGCNA), there was considerable overlap in genes associated with estradiol levels according to the two methods in the FOL phase.

### 3.5 Functional enrichment and transcription factor analyses of the most significant modules revealed pathways associated with epithelial barrier integrity and cell cycle processes

**Figure 4:**
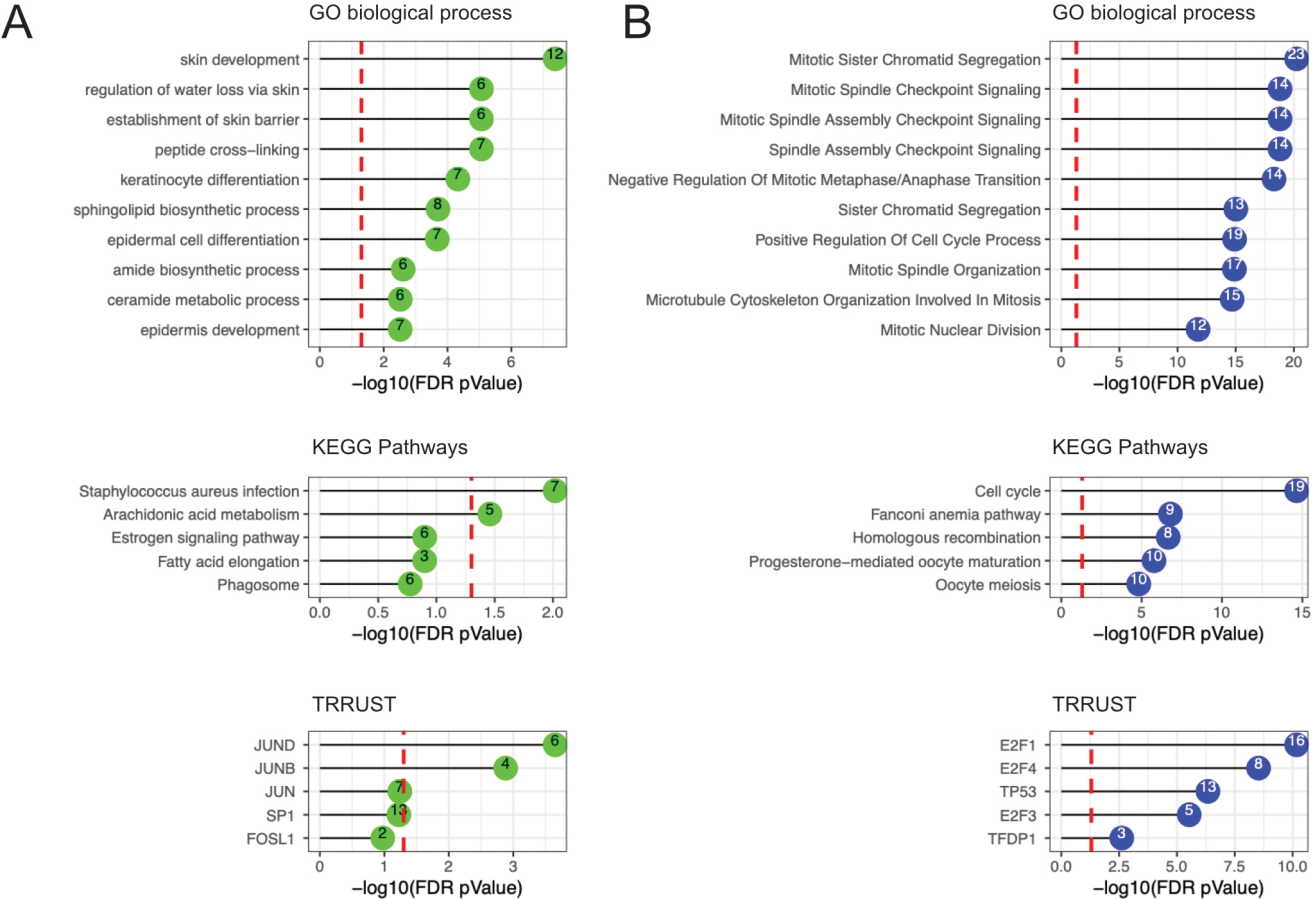
Functional enrichment results in the FOL phase using GO terms, KEGG pathways, and the TRRUST database. **(A)** Functional enrichment results for the green module (positively associated with estradiol). **(B)** Functional enrichment results for the blue module (negatively associated with estradiol). The number within the colored circles represents the number of genes in overlap between the identified term and the module. The GO terms and KEGG pathways represent biological pathways whereas the TRRUST represent transcription factors. The red dashed line represents the FDR-adjusted p-value cutoff of 0.05. FDR, false discovery rate; GO, gene ontology; KEGG, Kyoto Encyclopedia of Genes and Genomes; TRRUST, Transcriptional Regulatory Relationships Unraveled by Science-based Text.

To decipher biological functions, functional enrichment of genes included in the two significant modules identified from the FOL samples was analyzed in reference to the GO and KEGG databases (**Figure 4**; **Supplementary Table 7**). The green module (which was positively associated with estradiol levels) was associated with epithelial barrier structure and function, such as “skin development”, “establishment of skin barrier”, and “keratinocyte differentiation”. To explore the regulatory mechanism of gene expression in relation to the green module, transcription factor analysis was performed using the TRRUST database. The green module was significantly correlated with transcription factors JUND and JUNB, which together with FOSL1 are members of the dimeric activator protein-1 (AP1) complex. AP1 regulates several physiological processes, including epidermis tissue homeostasis and inflammation. The green module was also correlated with SP1, a transcription factor involved in cell differentiation and immune responses.

In contrast, the blue module (which was negatively associated with estradiol levels) was associated with GO and KEGG terms relating to cell cycle functions, such as “mitotic spindle checkpoint signaling”, “positive regulation of cell cycle process”, “microtubule cytoskeleton organization involved in mitosis”, and “cell cycle” (**Figure 4**; **Supplementary Table 7**). TRRUST database analysis revealed that the blue module was significantly associated with transcription factors E2F1, E2F3, and E2F4 (members of the E2F family). Significant associations were also found with the *TFDP1* gene, which codes for the heterodimeric partner DP1 of E2F. *E2F* genes are involved in activating and suppressing various biological functions, including cell cycle control. Significant associations were also found between the blue module and *TP53* gene expression. The transcription factor TP53 exerts multiple profound effects on cell cycle control and tumor suppression.

Overall, these data indicate that estradiol levels correlated positively with pathways involved in epithelial barrier structure and function, and negatively with pathways involved in cell cycle activities.

### 3.6 Estradiol was positively correlated with expression of epithelial barrier proteins in genital secretions

**Figure 5:**
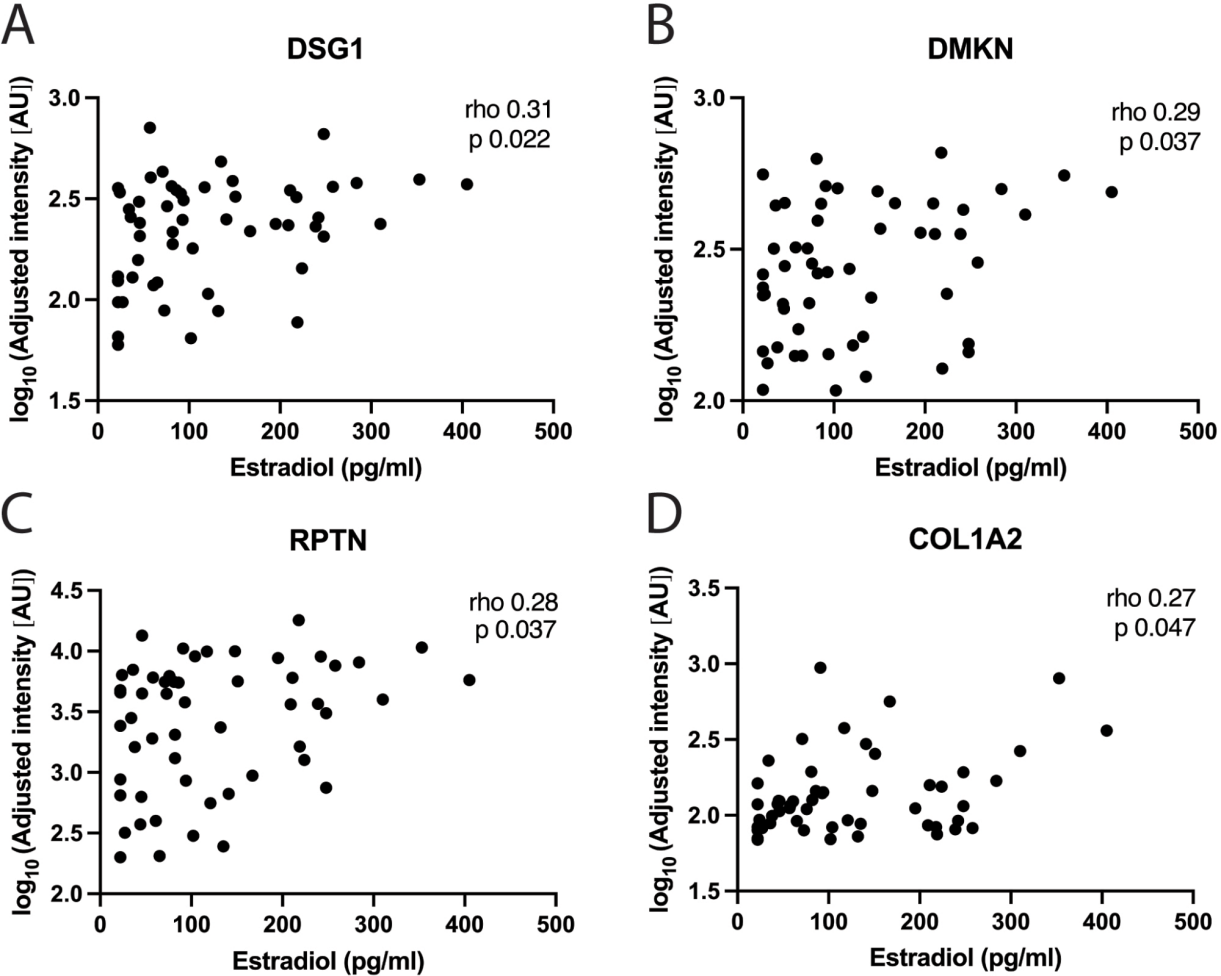
Correlations between estradiol and protein levels in genital secretions. Correlations between systemic estradiol levels and: **(A)** DSG1, **(B)** DMKN, **(C)** RPTN and **(D)** COL1A2, in CVL-samples using the protein profiling method at the FOL phase visit. Each data point represents one subject. Plasma estradiol levels are shown on the x-axis and the log_10_ adjusted intensity of each protein in AU on the y-axis. Correlation analyses were performed using Spearman’s correlation test. P-values <0.05 were considered significant. CVL, cervicovaginal lavage; AU, arbitrary units.

To complement RNA-sequencing results, antibody-based protein profiling targeting 74 proteins was performed on CVL samples. CVL samples represent proteins shed from both the upper and lower female reproductive tract. We analyzed 54 FOL samples (from the full cohort) and 49 LUT samples (44 from the full cohort used for RNA-sequencing and 5 additional samples). Clinical data for these participants are presented in detail (**Supplementary Table 8**). At the FOL phase visit, several epithelial barrier proteins, including DSG1 (r=0.31, p=0.022), DMKN (r=0.29, p=0.037), RPTN (r=0.28, p=0.037) and COL1A2 (r=0.27, p=0.048), were positively correlated with estradiol (**Figure 5**; **Supplementary Table 1**). Furthermore, at the FOL visit, higher levels of progesterone correlated with lower expression of CD5L, LSP1 and AHSG (**Supplementary Table 1**).

At the LUT phase visit, higher levels of estradiol only correlated with a lower expression of CSTA, as defined by one of the two antibodies analyzed (**Supplementary Table 1**). In contrast, progesterone levels during the LUT phase positively correlated with 9 of the 74 proteins, including innate immune proteins (e.g. NCF2, S100P, CXCL10 and LYZ) and epithelial structural proteins (e.g. KRT1 and KRT18) (**Supplementary Figure 5**; **Supplementary Table 1**).

Collectively, our data of these CVL samples indicate that higher levels of estradiol during the FOL phase correlated with increased expression of epithelial barrier proteins. This contrasted to the limited correlations of protein expression with estradiol in the LUT phase and with progesterone in the FOL phase. During the LUT phase, progesterone correlated with both innate immune and epithelial barrier proteins.

### 3.7 Estradiol was positively correlated with increased expression of DSG1 and a more stable ectocervical epithelial barrier as assessed *in situ*

**Figure 6:**
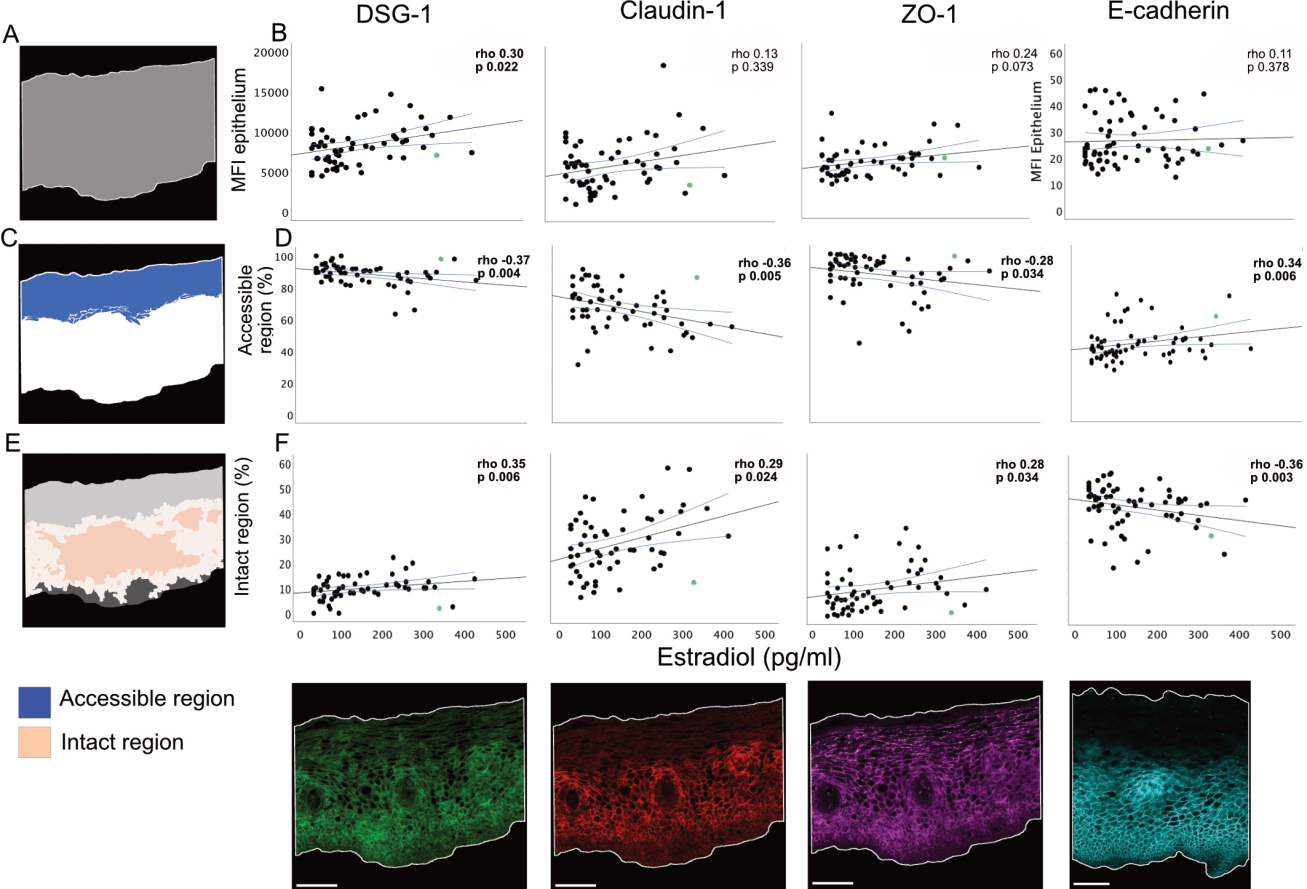
Correlations between estradiol and ectocervical integrity of epithelial junction proteins. **(A)** Schematic representation of the epithelial compartment. **(B)** Correlations between estradiol at the FOL phase visit and MFI in the epithelial compartment for DSG-1, Claudin-1, ZO-1 and E-cadherin. **(C)** Schematic representation of the theoretical accessible region for incoming or outgoing microorganisms. **(D)** Correlations between estradiol and coverage of accessible region for DSG1, Claudin-1, ZO-1 and E-cadherin. **(E)** Schematic representation of the intact region. **(F)** Correlations between estradiol and coverage of intact region for DSG1, Claudin-1, ZO-1 and E-cadherin. Below the correlation plots are illustrative images of each staining, derived from the median study participant according to MFI. Scale bar 100 µm. Graphs show Spearman correlation with a linear regression line and 95% confidence interval. P-values <0.05 considered significant and marked in bold. Each data point represents one subject. The individual scoring positive for *Chlamydia trachomatis* is marked in green. FOL, follicular; MFI, Mean fluorescence intensity.

To analyze the influence of estradiol on protein expression and ectocervical barrier integrity, *in situ* immunofluorescence staining was performed. This included staining of DSG1, claudin-1, ZO-1 and E-cadherin on the ectocervical tissue samples obtained during the FOL phase. Most EJPs form net-like structures across the epithelium which is crucial for blocking intercellular entry of invading microorganisms (53, 54). We previously reported correlations between plasma estradiol levels and the MFI for DSG1 and claudin-1 using data from a partially overlapping cohort and a prior bioimage analysis workflow (8). In the current study, we analyzed 59 samples (all from the full cohort) using triple staining for DSG1, claudin-1 and ZO-1 and an additional 6 samples (n=65 in total) using single staining for E-cadherin, employing a newly developed workflow (**Supplementary Tables 9, 10**). First, to assess protein expression levels, the MFI of each of the four EJPs was measured and compared against estradiol levels. A positive correlation was found for DSG1 vs. estradiol levels (r=0.3.8, p=0.022) (**Figure 6**). Next, the coverage of a theoretical “accessible region” for incoming microorganisms was assessed for each of the four EJPs (**Supplementary Figure 1**). As EJPs form net-like structures, the accessible region is here defined as the theoretical distance an incoming virus can penetrate the epithelium before being obstructed by connected, continuous protein strands (**Supplementary Figure 1**). Higher estradiol levels correlated with a smaller accessible region for DSG1 (r=–0.37, p=0.004), claudin-1 (r=–0.36, p=0.005), and ZO-1 (r=–0.28, p=0.034), and with a larger accessible region for E-cadherin (r=0.34, p=0.006) (**Figure 6**). Finally, we examined the coverage of “intact” region of each of the four EJPs. An intact region is here characterized as the size of the network of continuous protein strands which functions as a proxy for barrier stability and protection against incoming and outgoing pathogens (**Supplementary Figure 1**). Estradiol levels positively correlated with a larger intact region for DSG1 (r=0.35, p=0.006), claudin-1 (r=0.29, p=0.024), and ZO-1 (r=0.28, p=0.034), while a negative correlation was found for E-cadherin (r=–0.36, p=0.003) (**Figure 6**). No correlation was found between estradiol levels and the epithelial height (**Supplementary Table 2**).

Collectively, these findings demonstrate that higher estradiol levels during the FOL phase correlate with increased DSG1 expression and enhanced structural barrier integrity as measured for DSG1, claudin-1, and ZO-1, but not for E-cadherin.

### 3.8 Progesterone had a minor impact on ectocervical epithelial integrity as assessed *in situ*

Next, we evaluated the possible influence of progesterone on the ectocervical epithelium by applying the bioimage analysis workflow to the same set of FOL phase samples. No correlations were found between progesterone levels and the MFI for any of the four EJPs (DSG1, claudin-1, ZO-1, E-cadherin). Progesterone levels did however correlate positively with a smaller accessible region for DSG1 (r=–0.42, p<0.001) and claudin-1 (r=–0.27, p=0.043) (**Supplementary Table 2**). Higher levels of progesterone also correlated with a larger intact region for DSG1 (r=0.42, p<0.001) and ZO-1 (r=0.28, p=0.032), and with a smaller intact region for E-cadherin (r=–0.29, p=0.022) (**Supplementary Table 2**). No correlation was found between progesterone levels and the epithelial height (**Supplementary Table 2**). Collectively, these data indicate that progesterone had no effect on protein expression of selected EJPs as defined by MFI, and a smaller impact on epithelial integrity as compared to estradiol.

## 4 Discussion

In this comprehensive analysis of human ectocervical mucosal samples, we found that high systemic levels of estradiol during the FOL phase of the menstrual cycle are associated with gene markers of enhanced epithelial integrity in the tissue. These findings were confirmed at the protein level in both tissues and secretions, including higher intensity and a more stable tissue distribution of EJPs, including DSG1. In contrast, progesterone levels during the FOL phase and both estradiol and progesterone levels during the LUT phase, had a marginal effect on ectocervical gene expression. The estradiol-mediated enhancement of the epithelial barrier during the FOL phase has implications for the defense against STIs, which are highly prevalent in this cohort of pre-menopausal Kenyan female sex workers.

Using a gene correlation network analysis (WGCNA), we revealed two distinct modules of genes correlating with estradiol levels during the FOL phase. The first module was positively correlated with higher estradiol levels and contained genes (including *DSG1, CLDN17*, and *FLG*) associated with epithelial barrier structure and functions, such as cell adhesion. The second module was negatively correlated with higher estradiol levels and included several genes involved in cell cycle regulation. In a regression model, including estradiol, progesterone and BV status, we further confirmed that higher estradiol levels were positively correlated with an upregulation of *DSG1*, as well as other epithelial barrier genes, such as *KRT76* and *KRT6C*. By transcription factor analysis, the first module was associated with the *AP1* complex, which regulates tissue homeostasis and immune responses, whereas the second module was associated with transcription factors of the *E2F* family and the *TP53* gene. To confirm these findings at the protein level, imaging analysis was performed on corresponding ectocervical tissue samples representing the FOL phase. This revealed a significant positive correlation between estradiol levels and an enhanced structural barrier integrity as measured for the selected markers DSG1, claudin-1 and ZO-1, but not for E-cadherin. In contrast to these findings, previous human, animal, and experimental studies have shown limited influence of estradiol on claudin-1 expression in the genital tract mucosa (8, 11, 12, 55). A recent study of an engineered vagina reported that ZO-1 levels decreased after co-culture with estradiol (20). The effect of estradiol levels on E-cadherin expression varied in different model systems (6, 19, 21), highlighting the context and site-specific complexity of comparing such studies. Nevertheless, we here confirmed our gene expression data showing an estradiol-associated impact on the ectocervical epithelium by assessing the same samples at the protein level for selected EJPs.

In contrast to estradiol levels, progesterone levels in the FOL phase samples did not correlate with any specific modules defined by WGCNA and only correlated with three DEGs according to the regression analysis. At the protein level, as assessed in our *in situ* imaging analysis of the corresponding tissue samples, progesterone had a smaller impact on the panel of selected EJPs as compared with estradiol. To better understand these sex hormone-mediated effects, ectocervical tissue samples representing the LUT phase of the same women were also assessed. Similar co-expression patterns were observed between the two menstrual phases for estradiol levels, as indicated by our preservation analysis results. However, neither estradiol nor progesterone levels in the LUT phase samples correlated significantly with any specific modules as defined by WGCNA or DEGs according to regression analysis. Since both progesterone and estradiol levels are higher in the LUT phase compared to the FOL phase, the estradiol mediated effect seen in the FOL phase could have been counteracted or masked by progesterone-mediated effects. We also speculate that the limited or absent expression of hormone receptors (ER and PR) in the ectocervical epithelium during the LUT phase (3, 4) renders the mucosa non-responsive to the hormones. This could also explain the limited effects recorded for progesterone in the FOL phase, as the PR is nearly absent in the ectocervical mucosa also during this menstrual phase (3, 4). Studies on mucosal tissue samples from other sites of the female reproductive tract expressing high levels of the ER and PR, such as the uterine cervical transformation zone or endometrial mucosa (5, 56), are likely to reveal other gene and protein expression profiles as compared with the ectocervix.

We could also correlate the sex hormone levels with abundancy of secreted proteins in corresponding CVLs. Elevated estradiol levels were associated with increased levels of proteins crucial for epithelial barrier integrity and function in the FOL phase samples. In addition to verifying increased DSG1 levels, increased levels of RPTN, DMKN, and COL1A2 were also identified. RPTN is involved in cornified cell envelope formation (57). We previously demonstrated that *RPTN* gene (8) and protein (45) expressions were downregulated in women using the hormonal contraceptive depot medroxyprogesterone acetate (DMPA, a low-estradiol stage), and upregulated during the ovulatory menstrual phase (a high-estradiol stage). Furthermore, we demonstrated that fluctuations in RPTN between the ovulatory and LUT phases were amplified in women with low levels of cervicovaginal *Lactobacillus*, reflecting a complex interplay between cervicovaginal microbiome composition and the effects of female sex hormones (45). DMKN can act as a soluble regulator of keratinocyte differentiation (58), and its expression is reduced in hypoestrogenic DMPA users (7). Vaginal *DMKN* gene expression has been reported to increase substantially in postmenopausal women after treatment with estradiol (23) further showing that regulation of *DMKN* expression is highly dependent on estradiol. COL1A2, identified in both our protein profiling and regression analysis as positively associated with estradiol, is involved in extracellular-matrix remodeling and cell-matrix interactions, and collagen I is increased in the endocervix during the follicular phase (59). Overall, these data confirm the estradiol-mediated effects on DSG1 and other structural epithelial gene and protein markers that we recorded for the ectocervical tissue samples from the FOL phase. We could also confirm that the limited effect of progesterone during the FOL phase and estradiol during the LUT phase, as observed in the tissue samples, exhibited a similar pattern in these CVL samples. However, progesterone levels during the LUT phase positively correlated with both structural and innate immune proteins, likely reflecting the protein composition from both the upper and lower female reproductive tract that CVL represents.

DSG1 is a transmembrane protein of the desmosome, an epithelial junction structure that mechanically couples adjacent keratinocytes in the skin and mucosal epithelium (53, 54). Decreased DSG1 activity (secondary to reduced expression or genetic mutation) is highly associated with various human skin disorders (54, 60). Our findings of associations between high estradiol levels and an increased DSG1 expression are consistent with previous animal models (11, 13–16, 22, 61) and have implications for susceptibility to STIs. DSG1 downregulation in mice has been associated with increased genital transmission of herpes simplex virus type 2 (12), and an increased susceptibility to cell-associated HIV infection was seen in a humanized mouse model (16). Elevated DSG1 levels have been reported in genital secretions of women with a *Lactobacillus*-dominant cervicovaginal microbiome, highlighting the complex interaction between the microbiome, female sex hormones, and epithelial barrier integrity (15). Furthermore, gene expression of the desmosome proteins DSG3 and DSC2 varies according to cervicovaginal microbiome composition (36). We previously observed downregulation of DSG1 and upregulation of DSG2 in women receiving DMPA (8). This indicate that downregulation of one desmosomal protein may be associated with compensatory upregulation of other desmosomal proteins.

Our study has several limitations, including potential bias introduced by our post-sampling precautions. Following LUT sample collection, study participants were required to abstain from unprotected sexual intercourse. Therefore, the potential effects of sex work on the genital tract mucosa may not have been present at the time of FOL sampling. Another limitation is that the determination of the menstrual phases was based on the self-reported onset of the last menstrual cycle. Nevertheless, estradiol and progesterone plasma concentrations correlated well with the self-reported phases at the group level. Serum concentrations of hormonal contraceptive compounds were not formally measured to rule out potential residual effects from usage more than six months before the study commenced. It is also likely that our analyses did not account for other potential confounders, including the cervicovaginal microbiome composition. However, we did adjust for BV status in the regression analysis of transcriptomic data. Furthermore, RNA-libraries from the FOL and LUT phase samples were sequenced using different systems, although a new alignment and new counts were generated to more accurately compare the time points. Importantly, our findings can only be applied to non-pregnant, premenopausal women, as significantly different sex hormonal ratios occur during pregnancy and after menopause. Likewise, the study is limited to Kenyan female sex workers and cannot be directly generalized to other groups. Collectively, these limitations highlight the complexity of determining drivers of sex hormone-associated phenotypes in *in vivo* studies.

This study was conducted in a geographic area where STI prevention is highly relevant, and it included study participants at high risk for STIs. Unraveling the intricate relationship between fluctuating levels of sex hormones, context-dependent expression of sex hormone receptors, and the female reproductive tract mucosa is essential for developing precise interventions against these infections.

## Data Availability

The raw RNA-sequencing data and sociodemographic and clinical characteristics of the study participants cannot be held in a public repository because of the sensitive nature of such personal data. Requests for data access can be made to the Karolinska Institutet Research Data Office (contact via rdo@ki.se). Access will be granted if requests meet the requirements of the data policy. Processed count data for the FOL phase samples were previously published (8, 36) and can be accessed in the Gene Expression Omnibus public repository, SuperSeries ID GSE217237. Processed counts for the not previously published LUT samples and the new count data for the FOL samples after new alignment can also be accessed under the same SuperSeries ID (GSE217237). All scripts used for the RNA-sequencing analysis can be found on GitHub (https://zenodo.org/doi/10.5281/zenodo.11198470). The protein profiling data per sample, including hormone values, have been published for the follicular phase samples (8). Corresponding new data for the luteal phase samples, along with the follicular phase data, are summarized in the **Supplementary Material** (**Supplementary Table 1**). The processed *in situ*–based imaging data can also be found in the **Supplementary Material** (**Supplementary Table 2**). All scripts used for the *in situ*–based image analysis can be found on GitHub (10.5281/zenodo.8365617).
