# Supplementary material for: Estradiol-mediated enhancement of the human ectocervical epithelial barrier correlates with desmoglein-1 expression in the follicular menstrual phase
Source: Front Endocrinol (Lausanne). 2024 Oct 8;15:1454006. doi: 10.3389/fendo.2024.1454006 (PMC11493707; doi:10.3389/fendo.2024.1454006)
Supplement: Supplementary file 1 [file DataSheet1.docx]

Supplementary Figures

**S Figure 1.** Schematic workflow for bioimage analysis of ectocervical epithelial tissue sections (page 2).

**S Figure 2.** Correlation of hormone levels at the FOL visit and the LUT visit (page 3).

**S Figure 3.** Gene Co-expression Network Analysis reveals no clusters of genes correlated with systemic levels of estradiol or progesterone in the LUT phase (page 3).

**S Figure 4.** Heatmap including all differentially expressed genes associated with levels of estradiol (page 4).

**S Figure 5.** Correlations between progesterone and protein levels in genital secretions (page 5).


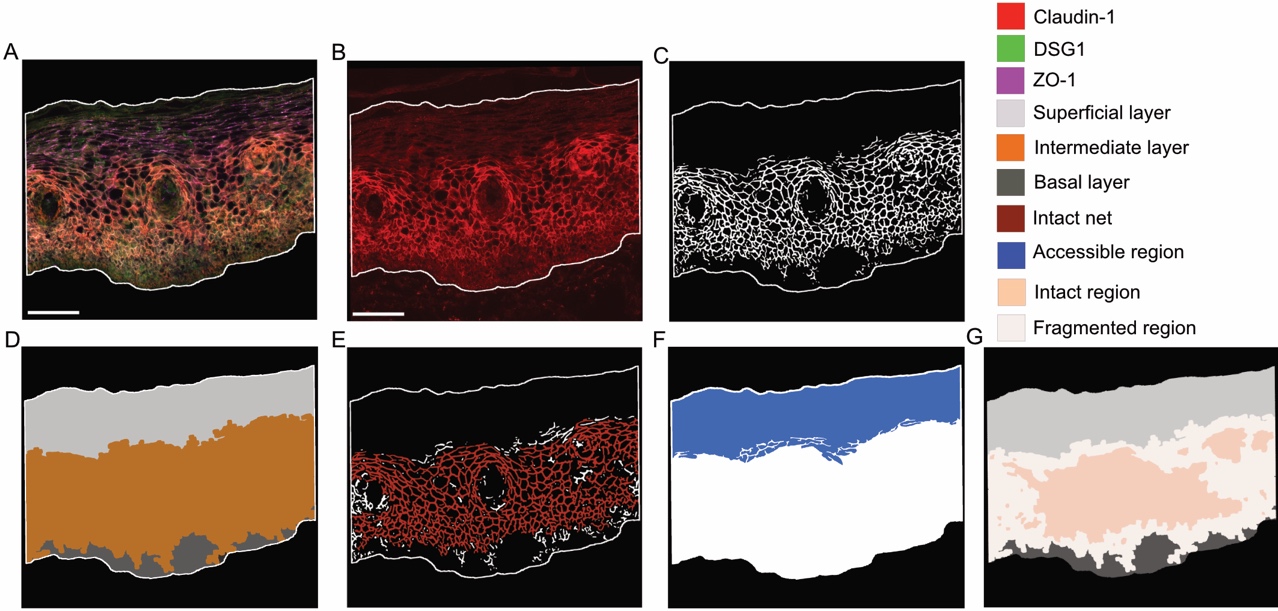


**S Figure 1. Schematic workflow for bioimage analysis of ectocervical epithelial tissue sections.** The epithelial compartment is marked with white lines (A-G). A) Combined image stained for Claudin-1 (red), DSG1 (green) and ZO-1 (purple). B) The immunofluorescent staining split into single channels, here represented by Claudin-1. C) The net-like structure of Claudin-1 in a digitalized format. D) As dictated by the net-like structure, the superficial layer (light grey), intermediate layer (yellow) and basal layer (dark grey). E) The net was classified as either intact (red) or fragmented (white) based on the connectivity of the protein strands. F) Assessment of the theoretical “accessibility” for incoming pathogens, as defined by the area lacking an intact net structure (blue). G) The intermediate layer was subdivided into an intact region (beige) and fragmented region (light beige) in reference to the integrity of the net.


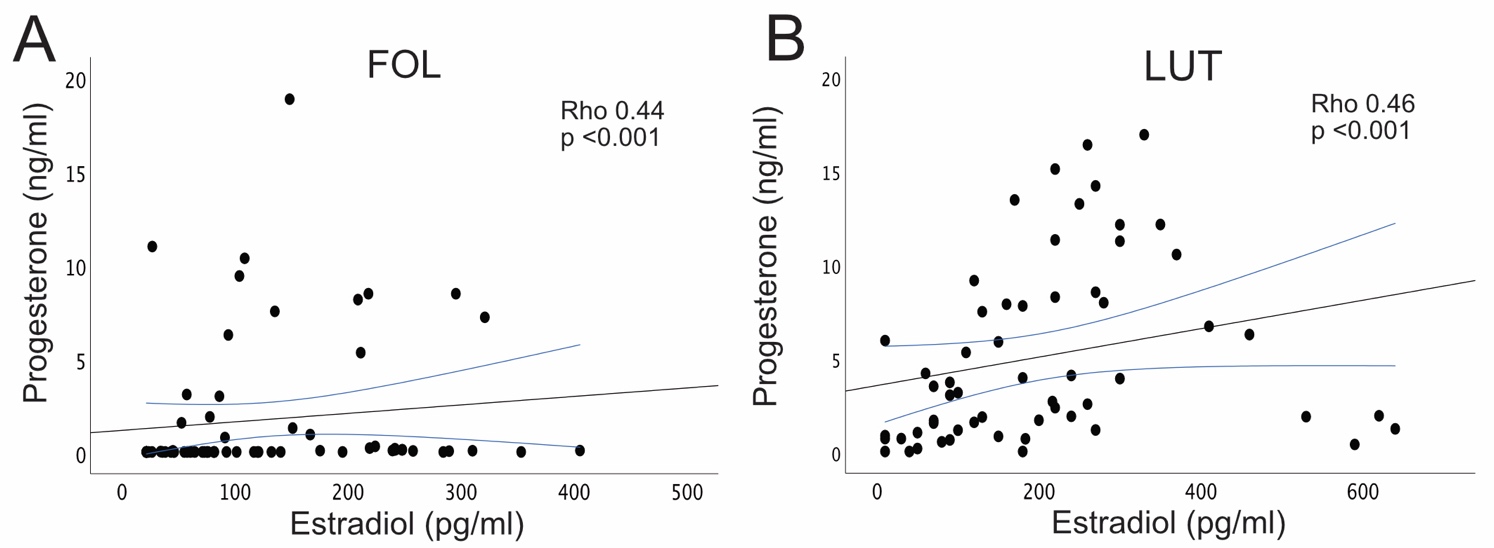


**S Figure 2. Correlation of hormone levels at the FOL visit and the LUT visit.**

Correlation analysis for the: A) FOL phase visit (n=66) and B) the LUT phase visit (n=58), for all samples included in the analysis.  Each dot represents one sample. Correlation coefficient (Rho) calculated by Spearman’s correlation test. Rho and p-values are shown in the right corner. The black line is a linear regression line and the blue lines represent the 95% confidence interval.

P-values <0.05 considered significant. LLD: lower limit of detection. FOL: follicular; LUT: luteal.

**S Figure 3. Gene Co-expression Network Analysis reveals no clusters of genes correlated with systemic levels of estradiol or progesterone in the LUT phase**.  The correlation between each module eigengene and the plasma levels of estradiol and progesterone in the LUT phase. The first value in each cell represents the Pearson correlation coefficient, and the value in parenthesis signifies the associated p-value. The strength of the association using Pearson correlation coefficient is also indicated by the colour of the cell, green colour representing a negative association and red colour a positive association with serum levels. LUT: luteal.


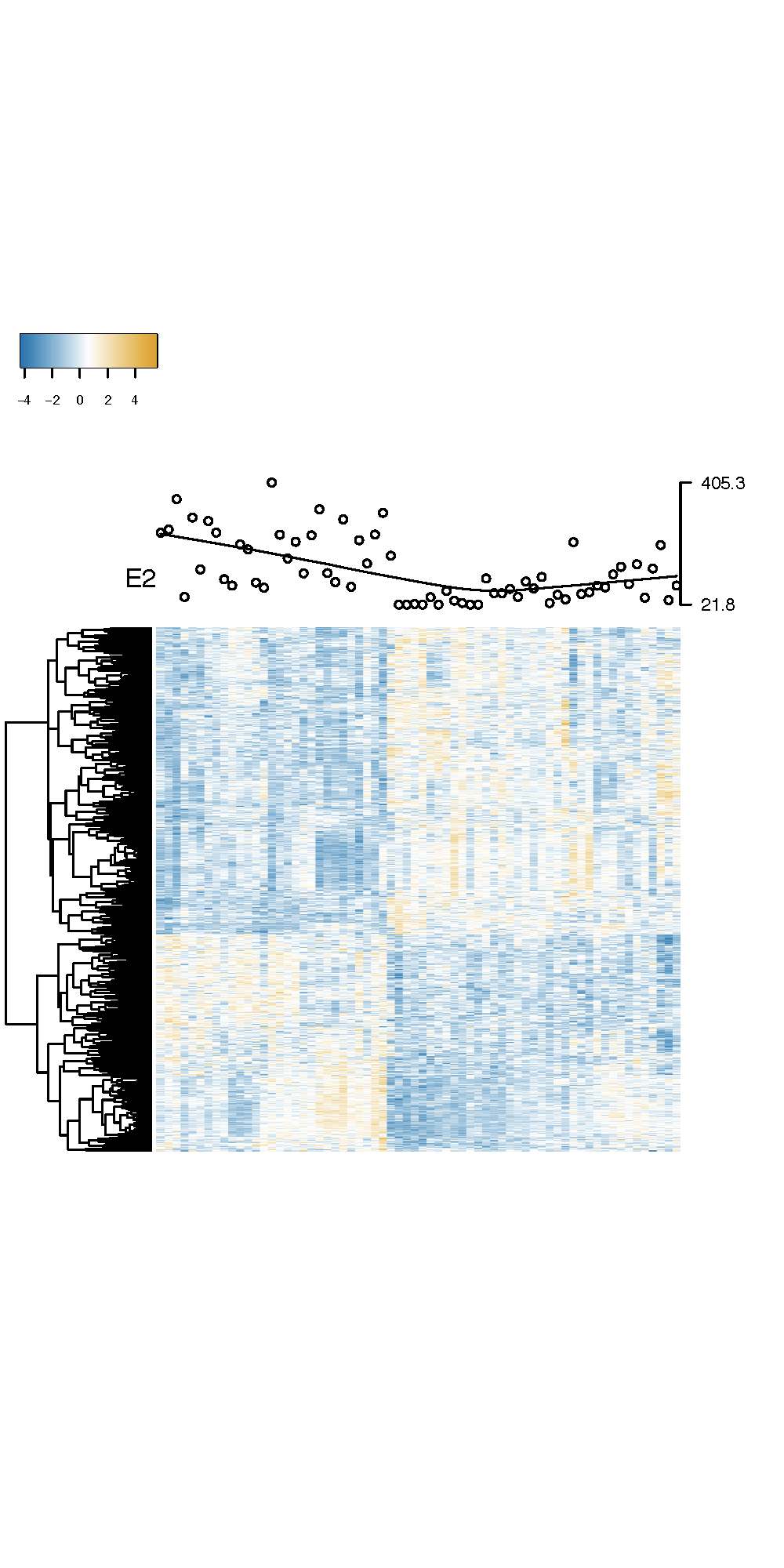


**S Figure 4. Heatmap including all differentially expressed genes associated with levels of estradiol.** Heatmap of all negatively (447) and positively (314) associated DEGs (FDR-adjusted P<0.05) with levels of estradiol at the FOL phase visit. Each gene is shown on the horizontal row and each sample on the vertical row. A blue colour represents a gene expression below average and a yellow colour a gene expression above average. FOL: follicular.


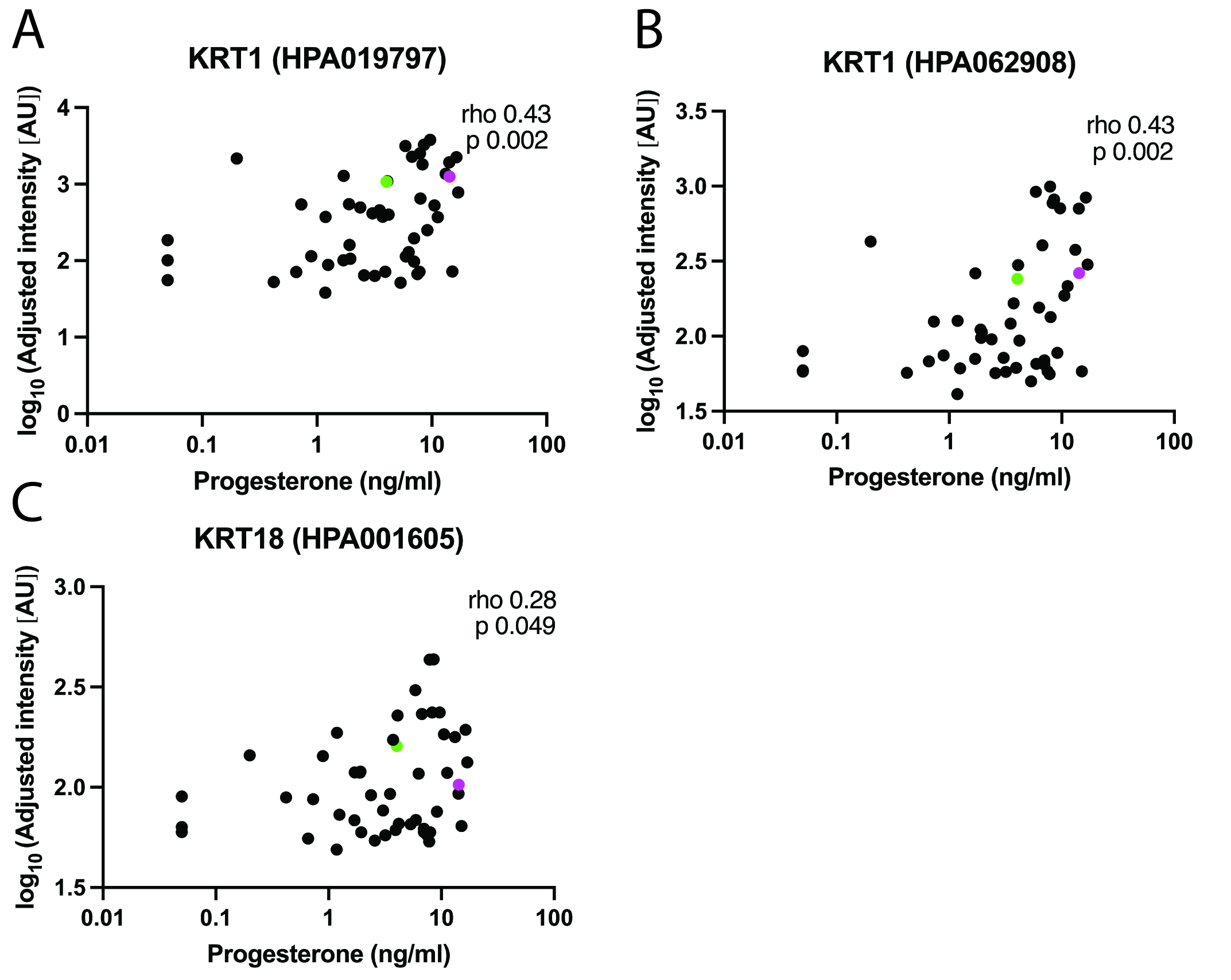


**S Figure 5. Correlations between progesterone and protein levels in genital secretions.** Correlations between plasma progesterone levels and A+B) KRT1 and C) KRT18 in CVL-samples using the protein profiling method at the LUT visit. Each data point represents one subject. Plasma progesterone levels are shown on the x-axis and the log_10_ adjusted intensity in AU of each protein on the y-axis. The name of the HPA-antibody is shown in parenthesis in the title. The individual with *Chlamydia trachomatis* is marked in green and the individual with *Neisseria gonorrhoeae* is marked in pink. Correlation analyses were performed using Spearman’s correlation test. P-values <0.05 were considered significant. CVL: cervicovaginal lavage; LUT: luteal; AU: arbitrary units; HPA: Human Protein Atlas.
