# Supplementary material for: Estradiol-mediated enhancement of the human ectocervical epithelial barrier correlates with desmoglein-1 expression in the follicular menstrual phase
Source: Front Endocrinol (Lausanne). 2024 Oct 8;15:1454006. doi: 10.3389/fendo.2024.1454006 (PMC11493707; doi:10.3389/fendo.2024.1454006)
Supplement: Supplementary file 9 [file Table8.docx]

Supplementary Material

| **S Table 8. Sociodemographic data and clinical characteristics of study subjects in the protein profiling assay.** | | |
| --- | --- | --- |
|  | **FOL (n=54)** | **LUT (n=49)** |
|  | Number or median (range or %) | Number or median (range or %) |
| **Age (years)** | 33 (20, 50) | 34 (21, 50) |
| **Months in sex work** | 36 (2, 372) | 36 (4, 372) |
| **Having a regular partner** |  |  |
| - Yes | 33 (61%) | 31 (63%) |
| **Years in school** | 10 (7,21) | 10 (7,21) |
| **Bacterial Vaginosis (BV; based on Nugent Score)** |  |  |
| - BV | 17 (31%) | 12 (24%) |
| - Intermediate | 13 (24%) | 12 (24%) |
| - Normal | 24 (44%) | 24 (49%) |
| - Not available | 0 (0%) | 1 (2%) |
| **Presence of STI*** | 0 (0%) | 1 CT, 1 NG (4%) |
| **Self-reported days since onset of last menses** | 9 (4, 44) | 21 (6, 26) |
| - Not available | 4 (7%) | 0 (0%) |
| **Plasma hormone levels** |  |  |
| *Estradiol (pg/ml)* | 92 (22, 405) | 220 (10,640) |
| - Below LLD** | 6 (11%) | 3 (6%) |
| *Progesterone (ng/ml)* | 0.05 (0.05, 19) | 4.1 (0.05, 17) |
| - Below LLD*** | 30 (56%) | 3 (6%) |

* Having an ongoing STI at time of enrolment (approximately 4 weeks prior to first sample visit) was an exclusion criterium for participating in the study. At each visit, testing was repeated for CT, NG, syphilis and *Trichomonas vaginalis*.

** LLD for estradiol was 20 pg/ml for the LUT visit and 22 pg/ml for the FOL visit.

*** LLD for progesterone was 0.9 ng/ml for LUT and 0.05 ng/ml for FOL visit.

STI: sexually transmitted infections. CT: *Chlamydia trachomatis*. NG: *Neisseria gonorrhoeae*. LLD: lower limit of detection
