# Supplementary material for: Estradiol-mediated enhancement of the human ectocervical epithelial barrier correlates with desmoglein-1 expression in the follicular menstrual phase
Source: Front Endocrinol (Lausanne). 2024 Oct 8;15:1454006. doi: 10.3389/fendo.2024.1454006 (PMC11493707; doi:10.3389/fendo.2024.1454006)
Supplement: Supplementary file 10 [file Table9.docx]

Supplementary Material

| **S Table 9. Sociodemographic data and clinical characteristics of study subjects included in the in-situ analysis for Claudin-1, ZO-1 and DSG1.** | |
| --- | --- |
|  | **FOL visit (n=59)** |
|  | Number or median (range or %) |
| **Age (years)** | 34 (20, 50) |
| **Months in sex work** | 72 (2, 372) |
| **Having a regular partner** |  |
| - Yes | 33 (56%) |
| **Years in school** | 10 (7, 21) |
| **Bacterial Vaginosis (BV; based on Nugent Score)** |  |
| - BV | 17 (29%) |
| - Intermediate | 16 (27%) |
| - Normal | 26 (44%) |
| **Presence of STI*** | 1 CT (2%) |
| **Self-reported days since onset of last menses** | 9 (3, 44) |
| - Not available | 4 (7%) |
| **Plasma hormone levels** |  |
| *Estradiol (pg/ml)* | 93 (22,405) |
| - Below LLD** | 6 (10%) |
| *Progesterone (ng/ml)* | 0.05 (0.05, 19) |
| - Below LLD*** | 33 (56%) |

* Having an ongoing STI at time of enrolment (approximately 4 weeks prior to first sample visit) was an exclusion criterium for participating in the study. At each visit, testing was performed for CT, NG, syphilis and *Trichomonas vaginalis*.
** LLD for estradiol was 22 pg/ml.
*** LLD for progesterone was 0.05 ng/ml.
STI: sexually transmitted infections. CT: *Chlamydia trachomatis*. NG: *Neisseria gonorrhoeae.* LLD: lower limit of detection.
